# The Long-Term Impact of Physical and Emotional Trauma: The Station Nightclub Fire

**DOI:** 10.1371/journal.pone.0047339

**Published:** 2012-10-15

**Authors:** Jeffrey C. Schneider, Nhi-Ha T. Trinh, Elizabeth Selleck, Felipe Fregni, Sara S. Salles, Colleen M. Ryan, Joel Stein

**Affiliations:** 1 Department of Physical Medicine and Rehabilitation, Spaulding Rehabilitation Hospital, Harvard Medical School, Boston, Massachusetts, United States of America; 2 Depression and Clinical Research Program, Department of Psychiatry, Massachusetts General Hospital, Boston, Massachusetts, United States of America; 3 Spaulding Rehabilitation Hospital, Boston, Massachusetts, United States of America; 4 Laboratory of Neuromodulation, Spaulding Rehabilitation Hospital, Harvard Medical School, Boston, Massachusetts, United States of America; 5 Department of Physical Medicine and Rehabilitation, University of Kentucky, Lexington, Kentucky, United States of America; 6 Sumner Redstone Burn Center, Department of Surgery, Massachusetts General Hospital, Boston, Massachusetts, United States of America; 7 Shriners Hospitals for Children®-Boston, Boston, Massachusetts, United States of America; 8 Division of Rehabilitation Medicine, Weill Cornell Medical College and New York-Presbyterian Hospital, New York, New York, United States of America; 9 Department of Rehabilitation and Regenerative Medicine, Columbia University Medical Center, New York, New York, United States of America; The University of Hong Kong, Hong Kong

## Abstract

**Background:**

Survivors of physical and emotional trauma experience enduring occupational, psychological and quality of life impairments. Examining survivors from a large fire provides a unique opportunity to distinguish the impact of physical and emotional trauma on long-term outcomes. The objective is to detail the multi-dimensional long-term effects of a large fire on its survivor population and assess differences in outcomes between survivors with and without physical injury.

**Methods and Findings:**

This is a survey-based cross-sectional study of survivors of The Station fire on February 20, 2003. The relationships between functional outcomes and physical injury were evaluated with multivariate regression models adjusted for pre-injury characteristics and post-injury outcomes. Outcome measures include quality of life (Burn Specific Health Scale–Brief), employment (time off work), post-traumatic stress symptoms (Impact of Event Scale–Revised) and depression symptoms (Beck Depression Inventory). 104 fire survivors completed the survey; 47% experienced a burn injury. There was a 42% to 72% response rate range. Although depression and quality of life were associated with burn injury in univariate analyses (p<0.05), adjusted analyses showed no significant relationship between burn injury and these outcomes (p = 0.91; p = .51). Post-traumatic stress symptoms were not associated with burn injury in the univariate (p = 0.13) or adjusted analyses (p = 0.79). Time off work was the only outcome in which physical injury remained significant in the multivariate analysis (p = 0.03).

**Conclusions:**

Survivors of this large fire experienced significant life disruption, including occupational, psychological and quality of life sequelae. The findings suggest that quality of life, depression and post-traumatic stress outcomes are related to emotional trauma, not physical injury. However, physical injury is correlated with employment outcomes. The long-term impact of this traumatic event underscores the importance of longitudinal and mental health care for trauma survivors, with attention to those with and without physical injuries.

## Introduction

Trauma is the direct personal experience of an event that involves actual or threatened death or serious injury. [1] Recent catastrophic events such as the wars in Afghanistan and Iraq, the 2011 Japanese tsunami, the September 11^th^ terrorist attacks, Hurricane Katrina and the 2010 Haiti earthquake have brought worldwide attention to the impact of trauma on peoples’ lives. The long-term sequelae of traumatic events include psychological, occupational, functional and quality of life impairments. [2], [3], [4], [5] Psychological trauma may accompany physical trauma or exist independent of it. However, the relative impacts of psychological and physical trauma are not well understood.

One of the deadliest fires in American history, The Station nightclub fire occurred on February 20, 2003 in West Warwick, Rhode Island. Pyrotechnic sparks ignited flammable sound insulation around the stage, creating a flash fire that engulfed the club in five minutes. Of the estimated 462 attendees, over 200 were injured and 100 died. [6] Video footage of the fire depicts stampeding patrons blocking the front entrance and the ensuing pandemonium as people tried to escape the burning building. The Station fire was an emotionally traumatic event for all survivors. In addition, a significant proportion of survivors also experienced physical burn injuries.

Burn injury is a form of physical trauma that results in well-documented long-term consequences such as occupational, [7], [8], [9] psychological [10], [11] and quality of life impairments. [12], [13] Similar to physical trauma, psychological trauma from a life-threatening event, such as a large-scale fire, can result in psychological impairments including post-traumatic stress disorder, major depression, anxiety disorders, [14], [15] as well as impairments in occupational, functional and quality of life outcomes. [16] Prior research has examined long term psychological outcomes after large fires and other non-fire disasters. [17], [18], [19] However, there is a paucity of longitudinal data on long term outcomes of trauma and, in particular, fires. Survivors of The Station fire are a unique cohort that enable us to differentiate the effects of physical and emotional trauma by examining outcomes of survivors with and without physical injury. To our knowledge, this is the first study to investigate the long-term effects of a large fire on its survivor population that includes both burned and nonburned survivors.

The purpose of this study is to (1) detail the multi-dimensional long-term effects of a catastrophic event, a large fire, on its survivor population and (2) assess differences in outcomes between survivors with and without physical injury in a multivariate analysis. The authors hypothesize that survivors with physical injuries will exhibit worse quality of life, psychological and employment outcomes compared to survivors without physical injuries when controlled for confounders and demographic characteristics.

## Methods

### Study and Survey Design

This study utilized a cross-sectional study design. All survivors present at The Station nightclub on the evening of the fire on February 20, 2003 were eligible for inclusion. There were no explicit exclusion criteria. All study procedures were approved by the Partners Human Research Committee.

Participants completed a survey of demographic, medical and outcome data. In addition, participants answered questions relating to occupational, legal, social and psychological status (Table 1). The questionnaire assessed the following outcomes: (1) quality of life, measured by the Burn Specific Health Scale – Brief (BSHS-B), (2) employment, measured by examining pre- and post-injury occupational history, (3) post-traumatic stress symptoms (PTSS), measured by the Impact of Event Scale – Revised (IES-R) and (4) depression symptoms, measured by the Beck Depression Inventory (BDI). The BSHS-B is a 40-item quality of life instrument that assesses nine domains, including heat sensitivity, affect, hand function, treatment regimens, work, sexuality, interpersonal relationships, simple abilities, and body image. Higher scores denote greater quality of life. It has established validity. [20] The IES-R is a 22-item self-report measure that assesses subjective distress caused by traumatic events. Higher scores correspond with a higher degree of PTSS; IES-R is not designed to diagnose post-traumatic stress disorder. It has established validity and reliability. [21], [22] The BDI is a 21-question self-report inventory that assesses the existence and severity of depression symptoms. Higher scores indicate more severe depression symptoms. It has established validity and reliability. [23], [24].

**Table 1:** Survey variables.

**Category**	**Variables**
Demographic	Age
	Gender
	Race
	Number of children
	Marital status
	Employment status
Social changes since fire	Married/engaged
	Divorced/separated
	Change of address
	Home adaptation
	Involvement in lawsuit
	Tobacco use
	Alcohol misuse (CAGE >0)
Medical	Total body surface area burned
	Body areas burned
	Hosptial length of stay
	Inhalation injury
	Inpatient rehabilitation
	Outpatient rehabilitation therapy
	Skin grafting
	Burn surgery
	Compression garment use
	Medical complications
Employment	Employment status pre-fire
	Employment status post-fire
	Return to same position
	Time off work
	Disability status
	Significant other time off work
	Significant other career change
Quality of life	Burn Specific Health Scale - Brief
	Numeric pain rating scale

### Recruitment

Subjects were recruited from June 2005 to October 2007 by (1) a letter from their treating rehabilitation physician, (2) survivor support group email listserve, (3) newspaper and radio advertisement, and (4) direct mailing. In the first wave of recruitment, the first three methods were utilized, as these were considered the least intrusive means of recruitment. At the time of the study, a local newspaper identified 330 likely survivors by name and hometown; the sources of information for these survivors were varied and included: survivors interviewed by the newspaper, survivors identified by other survivors, survivors identified by lawyers, survivors identified by relatives, survivors confirmed by hospitals, and survivors identified by photographers that took pictures in the nightclub. [25].

In the second wave of recruitment, a search agency was used to establish definitive mailing addresses for remaining likely survivors from the initial newspaper listing. The search agency encountered numerous difficulties identifying survivors that included: incomplete versions of survivors names (e.g., “J. Smith” could have been John Smith or Jay Smith), multiple contact addresses for one potential survivor (e.g., “John Smith from Main Street” versus “John Smith from Center Street”), and addresses with hometowns that differed from the newspaper listing (e.g. newspaper listed John Smith from Providence; search agency found John Smith from Portsmouth). In cases that did not have one definitive mailing address, such as the above scenarios, mailings were sent to each potential contact to attempt to reach as many survivors as possible.

The mailing to these remaining likely survivors included a brief explanation of the study and study staff contact information. Survivors were invited to notify study staff if they were or were not interested in the study. Interested survivors were provided the questionnaire, which was made available by email, password-protected website or mailed hard copy. If a completed survey was not received by two weeks, subjects received follow-up by email, phone, or mail in an effort to increase response rate. Subjects received monetary compensation for completing the survey ($25). Written informed consent was obtained for the subjects that were recruited by letter from their treating physician. For the remainder of the study subjects, a waiver of consent was obtained from the Partners Human Research Committee. The Common Rule [26] and HIPAA Privacy Rule [27] allow an Institutional Review Board to approve a waiver of informed consent for research when specific criteria are met. Identifying data was kept separate from the rest of the data and was not used in data analysis or reporting.

### Statistical Analysis

The response rates were calculated as the ratio of the number of survivors with completed surveys to the total number of eligible survivors. Given that the newspaper listing of likely survivors was only a rough estimate of the total number of survivors and the limitations of the search agency identification process, the exact number of eligible survivors is unknown. Therefore, two response rates were calculated to provide a range. For the minimum response rate calculation, eligible survivors were defined as all individuals with confirmed contact information as well as any mailings without a response; those returned because of wrong addresses were considered cases of unknown eligibility. For the maximum response rate calculation, eligible survivors were defined as only those with confirmed contact information; mailings without a response and those returned because of wrong addresses were considered cases of unknown eligibility. [28] Responders that completed the survey were compared to non-responder survivors using adjusted multivariate analysis with the following variables: gender, age and median home value by zip code. This analysis used the latter definition of eligible survivors.

The prevalence of quality of life impairments (BSHS-B), depression symptoms (BDI) and PTSS (IES-R) in the study population were compared with historical data of these outcomes in the general population. [29], [30], [31] BSHS-B scores were grouped into physical (items #1–9) and generic (#10–30) subscores for purposes of comparison. [29] A BDI score greater or equal than 13 was used as cutoff for depression. [30] The characteristics of the BSHS general population exhibited a mean age 40 years and 61% were female. For the BDI normative sample, the age range was 18–64 and 50% were female. [32] For the IES-R normative sample, the age range was 16–78 and 55% were female.

A multivariate model was used to assess the relationship between burn injury and outcomes (depression symptoms, PTSS, employment and quality of life). We adjusted these comparisons for the following independent pre-fire variables: age, gender, race, marital status, number of children, pre-fire employment; and outcomes: PTSS, depression symptoms, employment (time off work and employment status) and quality of life. The outcomes were included in the model because the outcomes are significantly correlated with each other, and it is critical to understand whether their relationship is independent of physical injury.

In the first step of modeling, univariate analyses were examined for each of the outcomes with burn injury as the main independent variable. Next, forward stepwise multivariate linear regression analyses were used for continuous outcomes: depression symptoms, PTSS and quality of life. Forward stepwise logistic regression analysis was used for the employment outcome time off work, dichotomized as greater than or less than or equal to six months. We forced the variables that are considered clinically important in the model regardless of their statistical significance in the univariate analysis. Statistical analyses were performed with STATA software, version 11 (StataCorp 2009).

## Results

### Responders

**Figure 1:**
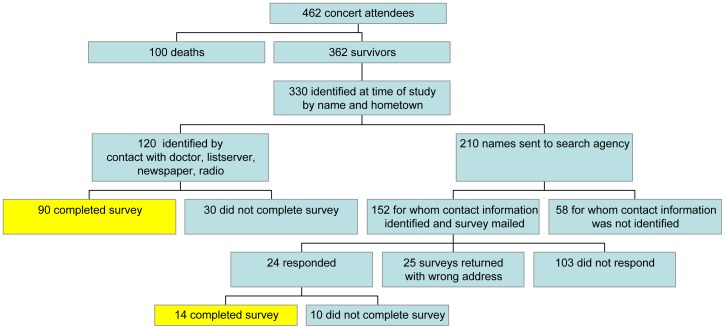
Flowchart of recruitment methodology.

104 of the 362 likely survivors completed the study survey. The minimum response rate calculation included 247 eligible survivors and the maximum response rate calculation included144 eligible survivors resulting in 42% and 72% response rates, respectively. (Figure 1) The first wave of recruitment resulted in the identification of 120 survivors. Of these, 90 survivors completed the survey. In the second wave, a search agency identified contact information for 152 potential subjects of the remaining 210 probable survivors. Of the 24 survivors that responded to the mailing, 14 completed the survey, six responded with interest in the study but did not complete the survey and four responded that they were not interested. There were 25 returned mailings because of wrong addresses and 103 mailings without a response. Of the 104 completed surveys, 74 were by password-protected website, 30 were by mailed hard copy and none were be email. Responders and non-responders were assessed for differences in socio-demographic characteristics. Multivariate analysis showed that gender and median home price by zip code were not significantly different between groups (age, p = 0.73; home price, p = 0.22). However, gender exhibited statistically significant differences between groups, with more males in the non-responder group (p = 0.05). We therefore adjusted our results to gender as to avoid a potential effect of non-responders in our results.

### Characteristics of study population

**Figure 2:**
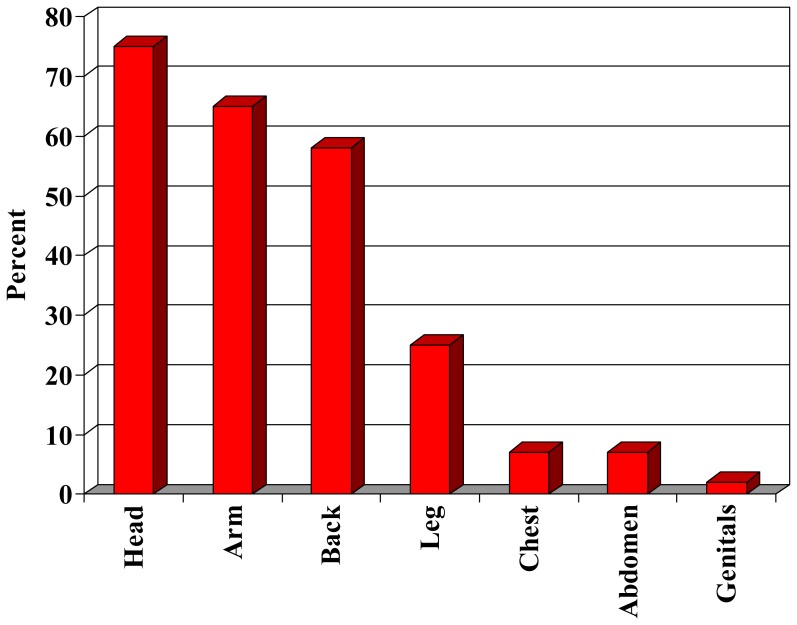
Body areas burned among survivors with burn injury.

Almost one-half of subjects experienced a burn injury as a result of the fire (47%). The characteristics of the two study groups were similar, except that survivors with burn injury were less frequently married and employed (p<0.05) (Table 2). Of those with burn injuries, the most common size burn was 1–20% total body surface area (59%). The head (75%) and arms (65%) were the most common areas burned (Figure 2). Respondents most commonly reported a hospital length of stay of 1–7 days (42%), followed by 1–5 months (30%). A minority of survivors with burn injuries reported an intensive care unit stay (43%) and inpatient rehabilitation stay (26%). Alcohol misuse was reported in 38% of survivors with burn injuries and 47% of survivors without burn injuries (the CAGE items were not administered to compare to pre-trauma status). In addition, multiple characteristics demonstrated no significant differences between injured and uninjured groups, such as self-reported psychiatric problems, social disruption (separation/divorce, change of address, home adaptation), alcohol and tobacco use, and counseling.

**Table 2:** Demographic and medical characteristics of study population.

Category	Survivors with Burn Injury	Survivors without Burn Injury
**Number of subject**s	49	55
**Male**, n (%)	28 (57)	36 (65)
**Age at injury**, mean years (sd)	32.1 (6.8)	32.6 (7.5)
**Race**, n (%)		
Caucasian	48 (98)	53 (96)
African American	0 (0)	0 (0)
Hispanic	1 (2)	1 (2)
Other	0 (0)	1 (2)
**Married or long-term partner**, n (%)*	15 (31)	35 (63)
**Employment status**, n (%)*		
Full-time	33 (72)	50 (92)
Part-time	7 (15)	2 (4)
Student	4 (9)	1 (2)
Unempoyed	2 (4)	1 (2)
**Children**, n (%)	26 (53)	23 (42)
**Total body surface area burned**, n (%)		
0–20%:	29 (59)	
21–40%:	13 (27)	
>40%:	7 (14)	
**Hospital length of stay**, n (%)		
1–7 days:	17 (42)	
1–3 weeks:	9 (23)	
1–5 months:	12 (30)	
6–12 months:	2 (5)	
**ICU stay**, n (%)	21 (43)	
**Inpatient rehabilitation stay**, n (%)	13 (26)	
**Outpatient rehabilitation therapy**, n, (%)	33 (67)	
**Psychosocial Characteristics**, n (%)		
Married or engaged	7 (15)	9 (16)
Divorced or separated	10 (21)	6 (11)
Change of address	23 (47)	28 (50)
Home adaptation	5 (10)	4 (7)
Started tobacco use	12 (24)	10 (18)
Alcohol misuse	19 (38)	26 (47)
Involvement in lawsuit *	47 (96)	18 (33)
Support group attendance*	14 (29)	2 (4)
Psychological counseling	30 (62)	32 (58)

* p-value ≤0.05; ^a^empty cells appear because survivors without burn injury do not have total body surface area burned, hospital length of stay, ICU stay, inpatient rehabilitation stay or outpatient rehabilitation therapy data.

### Outcomes

For PTSS, depression symptoms and quality of life the study cohort exhibited worse outcomes than population-based comparison groups. IES-R scores indexing PTSS for the study population was 28±22 (mean ± sd); this represented significantly more PTSS than the population-based comparison group (p<0.001). Depressive symptoms (BDI ≥13) were found in 35.4% of the study sample and this constituted a significantly larger proportion than controls (prevalence 4.2%; p<0.001). For quality of life, the study cohort exhibited mean BSHS-B physical and generic scores of 30±7 and 57±19, respectively. These scores also represented significantly worse quality of life than population-based control data (35±3, p<0.001; 71±14, p<0.001).

Survivors with and without burn injuries demonstrate significant impairments in quality of life, employment, PTSS and depression symptoms (Table 3). Unadjusted outcome data demonstrated that survivors with burn injuries exhibited lower quality of life scores compared to survivors without burn injuries. For occupational outcomes, survivors with burn injuries returned to the same job after the fire less often (69% vs 91%), were more likely to be unemployed after the fire (33% vs 10%), and were more likely to be on disability (29% vs 2%) than those without burn injuries. Survivors with burn injuries also reported more time off work than survivors without burn injuries. Survivors with and without burn injuries demonstrated similar levels of severe (35%; 21%), moderate (21%; 24%), mild (23%; 33%) and subclinical (21%; 22%) PTSS. Regarding depressive symptoms, the majority of survivors with burn injuries had minimal levels of depression (52%), followed by moderate (22%), mild (15%), and severe (11%); the vast majority of survivors without burns demonstrated minimal levels of depression (80%).

**Table 3:** Outcomes of study participants.

Outcomes and subcategories	Survivors with Burn Injury	Survivors without Burn Injury
**Quality of Life,** Burn Specific Health Scale – Brief, median (interquartile range)		
Sub-categories	Sub-scores	Sub-scores
Simple abilities	4 (3.7, 4)	4 (4, 4)
Hand function	4 (3.3, 4)	4 (4, 4)
Affect	2.9 (2.1, 3.7)	3.4 (2.6, 3.9)
Interpersonal	3.8 (3, 4)	4 (3.5, 4)
Sexuality	4 (3.2, 4)	4 (3.7, 4)
Body Image	3.5 (2.6, 4)	4 (4, 4)
Heat Sensitivity	2.8 (1.8, 3.6)	4 (4, 4)
Treatment regimes	3.8 (3.1, 4)	4 (4, 4)
Work	3.8 (3, 4)	4 (4, 4)
**Occupational Outcomes**, n (%)		
Return to same job post-fire	33 (69)	50 (91)
Employment after fire		
Full-time:	12 (45)	23 (80)
Part-time:	6 (22)	3 (10)
Unemployed:	9 (33)	3 (10)
Time off work		
1–7 days:	3 (7)	28 (56)
1–3 weeks:	3 (7)	14 (28)
1–5 months:	16 (35)	2 (4)
6–12 months:	12 (27)	0 (0)
>1 year:	11 (24)	6 (12)
Disability	14 (29)	1 (2)
**Post-Traumatic Stress Symptoms,** Impact of Event Scale-Revised, n (%)		
Subclinical	10 (21)	12 (22)
Mild	11 (23)	18 (33)
Moderate	10 (21)	13 (24)
Severe	17 (35)	11 (21)
**Depressive Symptoms,** Beck Depression Inventory, n (%)		
Minimal	24 (52)	42 (80)
Mild	7 (15)	2 (4)
Moderate	10 (22)	4 (8)
Severe	5 (11)	4 (8)

### Univariate and multivariate adjusted analysis

The results of the univariate and multivariate analyses are presented in Table 4. Although survivors with burn injuries exhibited lower mean quality of life scores (BSHS-B) compared to survivors without burn injuries in the univariate analysis (mean difference of 4.5 points, p<0.001), adjusted analysis showed no significant differences between the two groups (burn injury, beta coefficient =  −1.17, p = 0.51). Variables related to employment confounded the relationship between burn injury and quality of life. Similar results were found for depression symptoms (BDI); there was a statistically significant relationship with burn injury in the univariate analysis (p = 0.01) but not in the multivariate analysis (p = 0.91). In addition, variables associated with employment also confounded the relationship between burn injury and BDI scores. For PTSS (IES-R) there was no significant relationship with burn injury in the univariate and multivariate analyses (p = 0.13 and p = 0.79, respectively).

For the relationship between post-fire employment outcomes and burn injury, the variable time off work was used as this variable better indexed employment changes associated with burn injury than employment status (based on changes in the Beta coefficient of the independent variables). The relationship between time off work and burn injury was significant in the univariate and adjusted analyses (univariate analysis: p<0.001, OR = 7.6; adjusted analysis: p = 0.03, OR = 4.03).

**Table 4:** Univariate and multivariate analyses of outcomes.

	Outcomes										
	Quality of Life, Burn Specific HealthScale – Brief (BSHS-B)			Depressive Symptoms, Beck Depression Inventory (BDI)		Post-Traumatic Stress Symptoms, Impact of Event Scale-Revised (IES-R)			Employment, Time off work		
Variable	Coefficient (95% CI)		p-value	Coefficient (95% CI)	p-value	Coefficient (95% CI)		p-value	Odds Ratio (95% CI)		p-value
**Univariate analysis**											
Burn Injury		−4.50 (−6.45, −2.55)	<0.001	5.63 (1.31, 9.95)	0.01		6.61 (−1.90, 15.12)	0.13	7.67 (2.73, 21.56)		<0.001
Constant		33.66 (32.26, 35.07)	<0.001	8.52 (5.56, 11.48)	<0.001		25.24 (19.40, 31.08)	<0.001	–		–
**Multivariate analysis**											
Burn Injury		−1.17 (−4.79, 2.44)	0.51	−0.28 (−5.53, 4.96)	0.91		1.58 (−10.78, 13.94)	0.79		4.04 (1.11, 14.63)	0.03
Gender		−1.02 (−4.43, 2.40)	0.54	2.43 (−2.41, 7.27)	0.31		−4.97 (−16.46, 6.51)	0.38		0.83 (0.25, 2.77)	0.76
Age		0.01 (−0.23, 0.24)	0.94	−0.04 (−0.38, 0.30)	0.80		0.22 (−0.57, 1.01)	0.57		1.08 (0.98, 1.20)	0.12
Race		−8.29 (−18.45, 1.87)	0.10	−9.65 (−24.58, 5.27)	0.19		−2.63 (−39.17, 33.91)	0.88		0.44 (0.02, 12.71)	0.63
Marriage		2.45 (−1.17, 6.06)	0.18	3.59 (−1.60, 8.79)	0.17		−1.52 (−14.30, 11.25)	0.81		0.61 (0.18, 1.99)	0.41
Pre-fire employment		−0.68 (−5.13, 3.78)	0.76	−0.46 (−6.88, 5.96)	0.88		4.21 (−10.83, 19.25)	0.57		0.86 (0.19, 3.82)	0.84
Number of children		−0.19 (−1.72, 1.33)	0.80	−1.53 (−3.63, 0.57)	0.14		3.91 (−1.00, 8.82)	0.11		0.79 (0.46, 1.34)	0.38
Time off work		−2.34 (−5.98, 1.29)	0.20	−1.51 (−6.91, 3.88)	0.57		−1.85 (−14.64, 10.95)	0.77		–	–
Post-fire employment		−0.85 (−4.08, 2.39)	0.59	−0.81 (−5.48, 3.86)	0.72		2.14 (−8.88, 13.16)	0.69		–	–
BDI		−0.39 (−0.64, −0.14)	0.004	–	–		1.74 (1.05, 2.43)	<0.001		1.02 (0.92, 1.14)	0.66
IES-R		0.03 (−0.09, 0.16)	0.58	0.31 (0.19, 0.44)	<0.001		–	–		0.96 (0.92, 1.01)	0.14
BSHS-B		–	–	−0.80 (−1.32, −0.29)	0.004		0.40 (−1.06, 1.85)	0.58		0.87 (0.73, 1.03)	0.11
Constant		44.78 (32.01, 57.55)	<0.001	39.89 (11.37, 68.42)	0.008		−15.60 (−93.92, 62.72)	0.68		–	–

a In the univariate analysis an empty cell occurs because the logistic regression analysis for the binary outcome employment does not produce a constant. In the multivariate analysis empty cells occur where the outcome and variable are the same.

## Discussion

This is the first study to investigate the long-term effects of a large fire on its survivors. This cross-sectional examination exposes the profound multidimensional impact of The Station fire on its survivors. At long-term follow up, the lives of survivors with and without burn injuries were significantly altered in multiple arenas. Fire survivors experienced impairments in the four outcome measures studied: PTSS, depression symptoms, quality of life and employment. Additional survey variables reveal a complex picture of life disruption that includes lawsuits, occupational changes, alcohol misuse, tobacco use, divorce, supportive counseling, changes of address, hospitalizations, outpatient rehabilitation therapy, and issues with interpersonal abilities and sexuality. The authors plan continued follow up of this cohort that will provide additional understanding of the long-term sequelae of trauma as further improvements in outcomes are possible. [33].

Contrary to our initial hypothesis, survivors with and without physical injury exhibit no significant difference in three of the four outcomes (quality of life, PTSS and depression symptoms), after controlling for pre-fire and outcome variables. Survivors that experienced physical and emotional trauma (those with burn injuries) demonstrate the same outcomes as those that experienced emotional trauma alone (those without burn injuries). Our analysis suggests that non-physical trauma is the primary determinant of these outcomes. This is in contrast to literature documenting post traumatic stress, depression and quality of life impairments in burn survivors. [10]–[13] This finding underscores the overwhelming impact of non-physical trauma on long-term outcomes. Military combat provides an interesting comparison. Marines deployed to Iraq and Afghanistan that reported feeling in danger of death demonstrated the highest odds of reporting PTSS. Those who were shot or seriously wounded also demonstrated increased odds of PTSS, although at a lower rate. [34] In a separate study of war veterans, the presence of PTSS was not related to severity of injury. [35].

On the other hand, survivors with burn injuries experienced worse outcomes than survivors without burn injuries in employment. This finding suggests a compounded effect of physical and non-physical trauma on employment outcomes. Burn injuries result in multiple physical complications requiring hospitalization and rehabilitation such as contractures, bony abnormalities, neuropathy, impaired thermoregulation, altered metabolism, chronic pain and hypertrophic scarring. [36] In this study, survivors with physical injuries experienced significant burns that included a high incidence of face and arm burns, large burns, prolonged hospital stays, intensive care unit stays and inpatient rehabilitation. Such physical consequences compound the emotional impact of trauma and impact occupational performance.

In this study, survivors with and without burn injuries exhibited significant levels of psychological, social and employment impairments despite access to well-developed state of the art medical services. At the time of the fire, the infrastructure for delivering medical care was intact even though regional disaster procedures were underdeveloped. [37] This experience contrasts with the sparse resources that exist in wartime, in the developing world, or after natural disasters. It is possible that many of the survivors without physical injuries did not receive medical care or were not referred for appropriate mental health care. Underutilization of medical and mental health care is demonstrated in other populations of trauma survivors, such as combat veterans, [38] refugees [39] and community violence victims. [40].

There are a few limitations to the study worth noting. One limitation is the cross sectional design, which offers a snapshot of survivors at one point in time. Because subjects completed the questionnaire at different points in time, a comparison of long-term outcomes is affected by the prolonged recruitment period. [41] Additionally, data was obtained directly from participants by a self-report questionnaire potentially introducing a reporting bias. However, this form of data collection was selected to include survivors not treated by the medical system and at long-term follow-up. Also, a minority of probable survivors completed the survey introducing a potential selection bias. Still, responder and non-responder analysis demonstrated no significant differences in age and socioeconomic status (median home value by zip code). Furthermore, there were significantly more female than male responders; historically men exhibit lower survey response rates than women. [42] The characteristics of the general populations used to determine baseline BSHS-B, BDI and IES-R scores exhibited a similar number of male and female subjects, which also differed from the study cohort. Lastly, given the inability to confirm definitive contact information for many survivors, two different response rates were calculated. Unlike medical patient registries, for example, which offer a definitive cohort with exact patient names, contact information, and confirmed presence of disease or exposure, the newspaper listing from which the cohort was drawn was inexact. The newspaper identified survivors from different sources with varying levels of certainty and listed incomplete contact information. Guidelines for calculating response rates utilize different definitions of eligibility depending on the reliability of the data. [28] The authors chose to provide a range of response rates to provide the reader with a better understanding of this issue. In spite of these potential limitations, this unique cohort provides us with valuable insight into the long-term effects of both physical and emotional trauma.

Research on other traumatic mass casualty events has also shown non-physical trauma to have lasting impact on quality of life, depression symptoms, PTSS and employment. After the September 11^th^, 2001 terrorist attacks on the World Trade Center, fire fighters frequently (12%) developed PTSS. [43] Interestingly, Manhattan residents exhibited elevated rates of PTSS and depression symptoms after the attacks. Those living closer to the site of the attacks demonstrated three times the incidence of PTSS as those further away, and loss of possessions due to the event was a predictor of PTSS. [2] In addition, trauma sequelae are long lasting. Veterans of the wars in Iraq and Afghanistan exhibited significant levels of PTSS (41%) and reported difficulty with social functioning, productivity, community involvement, and self-care almost four years after returning home. [3] A year following Hurricane Katrina, almost one-quarter of evacuated hemodialysis patients experienced PTSS. [5].

In summary, survivors of this large-scale fire at The Station nightclub exhibit significant levels of life disruption at long-term follow up. The findings suggest that emotional trauma, not physical injury, determines the outcomes quality of life, PTSS and depression symptoms. The long-term impact of this traumatic event underscores the importance of longitudinal and mental health care for trauma survivors, with attention to those with and without physical injuries.
